# Psychosocial and clinical predictors of patient satisfaction with cancer care

**DOI:** 10.1016/j.jsps.2022.01.020

**Published:** 2022-01-29

**Authors:** Fahad D. Alosaimi, Futoon S. Alsaleh, Luluh Y. Alsughayer, Lamees A. Altamimi, Ibrahim A Alfurayh, Nashwa M. Abdel-Aziz, Khalid A. Alsaleh

**Affiliations:** a Psychiatry Department, King Saud University, Riyadh, Saudi Arabia; b College of Medicine, King Saud University, Riyadh, Saudi Arabia; c South Egypt Cancer Institute, Assiut University, Assiut, Egypt; d Haematology-Oncology Center, King Saud University, Riyadh, Saudi Arabia

**Keywords:** Satisfaction with cancer care, Radiotherapy, Chemotherapy, Hormonal, Biological therapy, Depression, Anxiety, Social support, Saudi Arabia

## Abstract

**Purpose:**

Patient satisfaction with healthcare was recognized as an indispensable component of healthcare quality assurance programs for decades. Limited research has explored psychosocial variables impacting patient satisfaction with cancer care. The objective of our study was to identify the level of patient satisfaction with cancer care in Riyadh, Saudi Arabia and determine the psychosocial and clinical predictors of patient satisfaction.

**Methods:**

A cross-sectional observational study was carried out in 2018–2019 with patients with cancer at the Outpatient Oncology Clinic at King Saud University Medical City in Riyadh, Saudi Arabia. The questionnaire contained a visual analog scale (VAS) of satisfaction with cancer care, a VAS of satisfaction with social support, the Patient Health Questionnaire-9 Depression scale, and the Generalized Anxiety Disorder 7-item scale.

**Results:**

Out of the 400 patients approached, 280 agreed to participate in the study. Of the 280 patients participating in the study, 65% were satisfied with cancer care. Higher satisfaction was associated with being non-Saudi, being employed, having fewer household residents (≤4), being satisfied with social support, not receiving radiotherapy, and receiving hormonal or biological therapy. Having anxiety or depression was also associated with lower satisfaction. After adjustment for sociodemographic and clinical characteristics, being satisfied with social support, having ≤ 4 household residents, receiving hormonal therapy, and receiving biological therapy rather than radiotherapy were all independent predictors of higher satisfaction with cancer care.

**Conclusion:**

This study found an inadequate level of patient satisfaction with cancer care. Higher levels of satisfaction were associated with being satisfied with social support, using biological and hormonal therapy, while lower satisfaction was associated with a larger number of household residents (>4), depression, anxiety and using radiotherapy.

## 1 Introduction

Patient satisfaction with healthcare was recognized as an indispensable component of healthcare quality assurance programs by the World Health Organization for decades (Darby et al., 2003). Patient satisfaction is as an important indicator of the quality of healthcare systems (Aharony and Strasser, 1992). The knowledge attained from patient satisfaction scales can greatly benefit healthcare providers in identifying areas of improvement and appreciating patients’ needs and, consequently, developing more effective and better-quality services (Batbaatar et al., 2017), which ultimately leads to the optimization of management plans (Jackson et al., 2001). In addition, staff of healthcare centers can use the results of patient satisfaction scales to compare their own healthcare centers to others (Gupta, 2009).

Patients with cancer diagnoses face physical, psychological, social, educational and spiritual challenges (Carlson et al., 2004, Kaasa and Loge, 2003). Determinants of the satisfaction of such patients have been investigated and correlated with the healthcare setting, number of staff members, physician behavior and skills of the medical staff (Gupta, 2009). However, limited research has explored psychosocial variables impacting patient satisfaction. A study conducted with patients with ENT and GI cancer demonstrated that the overall predictors of satisfaction were young age, female gender, and greater attention to how the patient copes with his or her illness, as well as greater staff attention to the patient’s feelings about the diagnosis and psychosocial issues (Walker et al., 2003).

A similar study was performed on breast cancer patients from four Canadian cancer centers. Patient satisfaction was shown to be related to many factors, with significantly higher satisfaction found for older patients, those with smaller primary tumors and those with longer consultation times. Additionally, other factors significantly predicted greater patient satisfaction, including smaller tumor size, lack of patient assertiveness during the treatment consultation, and a consultation with a radiation oncologist rather than medical oncologist (Hack et al., 2010).

In Saudi Arabia, a single institution hospital-based study found 73% patients were satisfied with the cancer services provided (Sait et al., 2014), while another study conducted among palliative cancer patients, found emotional function to be more closely associated with overall satisfaction with care than physical function or global health status (Aboshaiqah et al., 2016). Although psychological variables have been examined well in relation to coping with cancer, few studies have examined in details the impact of psychosocial variables on patient satisfaction with cancer care. Our study aimed to identify the level of patient satisfaction with cancer care in Riyadh, Saudi Arabia and to examine the association of sociodemographic, clinical and psychosocial variables, including depression, anxiety and social support with patient satisfaction with cancer care.

## 2 Material and methods

This cross-sectional study was conducted at outpatient oncology clinics at the oncology center at King Saud University Medical City (KSUMC) in Riyadh, Saudi Arabia. KSUMC is a governmentally funded teaching medical city that provides free secondary and tertiary care services to patients from everywhere in Saudi Arabia. Qualified data collectors and nurses at the oncology clinic enrolled patients attending appointments during 2018–2019.

Through convenient sampling, adult patients with breast cancer, colon cancer, or lymphoma, which are the most common cancers encountered in the clinic, were included in this study, after they have signed an informed consent form. The study questionnaire contained a Visual Analogue Scale (VAS) of satisfaction with cancer care; questions on sociodemographic background, psychiatric history, and medical history; a VAS on satisfaction with social support; the Patient Health Questionnaire-9 (PHQ-9) Depression scale; and the Generalized Anxiety Disorder 7-item (GAD-7) scale.

A VAS is a horizontal line of 10-mm, numbered from 0 to 10. VASs have been frequently used for the self-assessment of many parameters, including satisfaction (Blanchard et al., 1990, McCormack et al., 1988). It has been found to be very brief, less vulnerable to bias from confounding factors and avoided the ceiling effect (Voutilainen et al., 2016). A VAS score of>8 was considered “satisfied with cancer care” in this study, as calculated using the 75% quartile, in line with previous studies (Kawai et al., 2006, Onat and Kizilkaya Beji, 2012). Also, the patients were requested to define their level of satisfaction towards social support on another VAS where the cut-off of 8 was appraised by the 75% quartile and considered ‘satisfied with social support’ (Athanasou, 2019).

The PHQ is an efficient, reliable, and highly acceptable tool for screening depression, anxiety, and somatic disorders (Spitzer et al., 1999). The Arabic version of the PHQ was found to be valid and reliable in screening for many psychiatric disorders, including depression (PHQ-9) and anxiety (GAD-7) (AlHadi et al., 2017). Both the PHQ-9 and GAD-7 are highly specific and sensitive, with a cutoff score ≥ 10 for detecting depression and anxiety, respectively (Manea et al., 2012, Plummer et al., 2016). Cutoff points of 5, 10, and 15 indicate mild, moderate, and severe anxiety on the GAD-7, respectively, which are similar to the levels of depression on the PHQ-9 (Kroenke et al., 2001, Spitzer et al., 2006). To assess for suicidality, Item 9 of PHQ-9 was used, which asks about, “thoughts that you would be better off dead, or of hurting yourself in some way?” Patients may respond with the following answers: “not at all” (scoring zero), “several days” (scoring one), “more than half the days” (scoring two) or “nearly every day” (scoring three). Patients who scored one or more on Item 9 were labeled positive suicidal ideations (Walker et al., 2008).

### 2.1 Statistical analysis

Statistical analysis was performed with JMP Pro software version 14.0 (SAS, Cary, NC). Continuous variables are presented as the means and standard deviations (SDs). Categorical variables are reported as proportions. A *t*-test or a Wilcoxon rank sum test was used to evaluate continuous variables, and a chi-square test (χ2) or Fisher’s exact test was used to test categorical data. Univariate analysis of predictors of patients being “highly satisfied with cancer care” was performed, and P values of < 0.2 were used to determine significance. Significant variables (P values of < 0.2) were included in a logistic regression model that was used to predict high satisfaction with cancer care. All tests were two-sided, and a p-value of < 0.05 was considered significant in the logistic regression model. The 75% quartile for satisfaction with cancer care was 8. Patients were labeled “highly satisfied” if they had a score > 8. The number of household residents was divided into 3 groups (≤4, 4–8, and > 8 residents) based on the interquartile range, as was age (15–42, 43–60, and > 60 years).

## 3 Results

We approached four hundred qualified patients; two hundred and eighty agreed to participate. The patients had a mean age of 51.8 years (SD of 14.3). Most patients were Saudis (84%), and 63% were female. The majority of the patients were married (81%); most were not employed (73%), and 40% had a family income of ≤ 5000 Saudi Riyals (SR). A significant proportion of the patients had at least a high school degree (59%), and the central region was the most common region of residence (80%). Having more than four household members was common (71%), and most patients owned their place of residence (71%).

The demographic characteristics according to scores of patients’ satisfaction with cancer care are displayed in Table 1a and Table 1(b). One hundred eighty-three patients (65% (183/280)) had satisfaction with cancer care scores > 8 (highly satisfied), while 35% (98/280) scored ≤ 8. Higher satisfaction was more common in non-Saudis than Saudis *(P* = 0.002). Additionally, fewer patients in the highly satisfied group owned their place of residence than rented (*P* = 0.01). Moreover, the highly satisfied group had more employed patients than the not highly satisfied group (31% (87/280) vs. 22% (62/280)) (*P* = 0.1). Finally, more highly satisfied patients had ≤ 4 household residents than not highly satisfied patients (35% vs. 16%, *P =* 0.002). Age, gender, marital status, family income, education, and region of residence did not differ between the highly satisfied and not highly satisfied groups.

**Table 1a:** Baseline sociodemographic characteristics and satisfaction with cancer care among patients in Saudi Arabia (N = 280).

Variable	Satisfied n = 183 (%)	Not Satisfied n = 97 (%)	Total n = 280 (%)	*P* value
**Age:** Mean (SD) 15–42 43–60 >60	52.5 (14.1) 36 (20%) 94 (51%) 53 (29%)	50.7 (14.5) 24 (25%) 47 (48%) 26 (27%)	51.8 (14.3) 60 (21%) 141 (50%) 79 (28%)	0.30 0.60
**Gender:** Male Female	68 (37%) 115 (63%)	35 (36%) 62 (64%)	103 (37%) 177 (63%)	0.80
**Nationality:** Saudi Non-Saudi	146 (80%) 37 (20%)	90 (93%) 7 (7%)	236 (84%) 44 (16%)	**0.002**
**Residency:** Owned Rented	121(66%) 62 (34%)	77(79%) 20 (21%)	198 (71%) 82 (29%)	**0.01**
**Employed:** Yes No	56 (31%) 127 (69%)	21 (22%) 76 (78%)	77 (27%) 203 (73%)	**0.10**
**Education:** Below high school High school Bachelor Master PhD	65 (36%) 41 (22%) 64 (35%) 9 (5%) 4 (2%)	33 (34%) 27 (28%) 34 (35%) 2 (2%) 1 (1%)	98 (35%) 68 (24%) 98 (35%) 11 (4%) 5 (2%)	0.50

**Abbreviations**: SD, standard deviation.

**Table 1b:** Baseline sociodemographic characteristics and satisfaction with cancer careamong patients in Saudi Arabia (N = 280).

Variable	Satisfied n = 183 (%)	Not Satisfied n = 97 (%)	Total n = 280 (%)	*P* value
**Number of household residents:** Mean (SD) ≤4 >4–8 >8	5.8 (2.8) 65 (35%) 89 (49%) 29 (16%)	6.6 (2.6) 16 (16%) 62 (64%) 19 (20%)	81 (29%) 151 (54%) 48 (17%)	**0.03** **0.002**
**Marital status:** Married Single Divorced Widowed	147 (80%) 19 (10%) 9 (5%) 8 (4%)	80 (83%) 12 (12%) 4 (4%) 1 (1%)	227 (81%) 31 (11%) 13 (5%) 9 (3%)	0.30
**Family income:** <5000 SR 5000–10,000 SR 10,001–15,000 SR 15,001–20,000 SR ≥20,000 SR	80 (44%) 37 (20%) 37 (20%) 16 (9%) 13 (7%)	32 (33%) 25 (26%) 18 (19%) 15 (15%) 7 (7%)	112 (40%) 62 (22%) 55 (20%) 31 (11%) 20 (7%)	0.25
**Region of residence:** Central South West North East	146 (80%) 15 (8%) 6 (3%) 8 (4%) 8 (4%)	78 (80%) 4 (4%) 9 (9%) 5 (5%) 1 (1%)	224 (80%) 19 (7%) 15 (5%) 13 (5%) 9 (3%)	0.80

**Abbreviations**: SD, standard deviation; SR, Saudi Riyals.

The patients’ clinical characteristics are displayed in Table 2. Breast cancer was the most common type of cancer (44%, 123/280). Most patients had received chemotherapy as part of their treatment (85%, 238/280). Very few patients were evaluated for a concurrent psychiatric illness (9%; 25/280); those who were evaluated were most commonly diagnosed with depression. A smaller proportion of patients in the highly satisfied group than in the not highly satisfied group had radiotherapy (26% (72/280) vs. 38% (106/280)) (*P* = 0.03). On the other hand, more highly satisfied patients than not highly satisfied patients had hormonal and biological therapy (14% (39/280) vs. 7% (19/280) (P = 0.07) and 14% (39/280) vs. 8% (22/280) (*P =* 0.1), respectively). Meanwhile, the correlations between satisfaction and chemotherapy (*P =* 0.3) and surgery (*P =* 0.8) were statistically insignificant.

**Table 2:** Clinical characteristics and satisfaction with cancer care among patients in Saudi Arabia (N = 280).

Variable	Satisfied n = 183 (%)	Not Satisfied n = 97 (%)	Total n = 280 (%)	*P* value
**Cancer type:** Breast Colon Lymphoma	77 (42%) 75 (41%) 31 (17%)	45 (46%) 36 (37%) 16 (17%)	122 (44%) 111 (40%) 47 (16%)	0.70
**Cancer stage:** 1 2 3 4	16 (18%) 31 (34%) 26 (29%) 18 (20%)	8 (18%) 15 (34%) 11 (25%) 10 (23%)	24 (17%) 46 (34%) 37 (27%) 28 (21%)	0.96
**Treatment:** Chemotherapy Surgery Radiotherapy Hormonal therapy Biological therapy	159 (87%) 96 (52%) 47 (26%) 26 (14%) 26 (14%)	80 (82%) 50 (52%) 37 (38%) 7 (7%) 8 (8%)	239 (85%) 146 (52%) 84 (30%) 33 (12%) 34 (12%)	0.30 0.80 **0.03** **0.07** **0.10**
**Visited a psychiatrist:** Yes No	15 (8%) 168 (92%)	10 (10%) 87 (90%)	25 (9%) 255 (91%)	0.50
**Diagnosed previously with a psychiatric disease:** Yes Depression Adjustment disorder Stress disorder Anxiety Not specified No	14 (8%) 9 (64%) 1 (7%) 1 (7%) 0 (0%) 3 (21%) 169 (92%)	5 (5%) 3 (60%) 1 (20%) 0 (0%) 1 (20%) 0 (0%) 2 (95%)	19 (7%) 12 (63%) 2 (11%) 1 (5%) 1 (5%) 3 (16%) 261 (93%)	0.40
**Using psychiatric medications:** Yes No	12 (7%) 171 (93%)	6 (6%) 91 (94%)	18 (6%) 262 (94%)	0.90

**Abbreviations**: SD, standard deviation.

The current patients’ psychological characteristics are shown in Table 3. Depression and anxiety were less common in the highly satisfied group than in the not highly satisfied group (15% (42/280) vs. 28% (78/280) (P = 0.01) and 19% (53/280) vs. 28 (78/280) (*P* = 0.07), respectively). As expected, patients who were highly satisfied with cancer care were also more highly satisfied with social support (score > 8) than patients who were not highly satisfied (81% (226/280) vs. 57% (160/280)) *(P* < 0.001).

**Table 3:** Psychological characteristics and satisfaction with cancer care among patients in Saudi Arabia (N = 280).

Variable		Satisfied n = 183 (%)	Not Satisfied n = 97 (%)	Total, n = 280 (%)	*P* value
**Depression** **(PHQ-9)**	Mean Score (SD): Not depressed (0–9) Depressed (>9) Minimal (0–4) Mild (5–9) Moderate (10–14) Moderately severe (15–19) Severe (20–27)	5.2 (4.4) 155 (85%) 28 (15%) 94 (51%) 61 (33%) 20 (11%) 7 (4%) 1 (1%)	7.4 (5.6) 70 (72%) 27 (28%) 29 (30%) 41 (42%) 13 (13%) 10 (10%) 4 (4%)	5.9 (4.9) 225 (80%) 55 (20%) 123 (44%) 102 (36%) 33 (12%) 17 (6%) 5 (2%)	**0.001** **0.01** **0.001**
**Suicidal ideation: item 9 of PHQ-9 ≥ 1**		11 (6%)	11 (11%)	22 (8%)	**0.10**
**Anxiety:** **(GAD-7)**	Mean score (SD): Not anxious (0–9) Anxious (>9) Minimal (0–4) Mild (5–9) Moderate (10–14) Severe (15–21)	4.6 (5) 149 (81%) 34 (19%) 110 (60%) 39 (21%) 21 (12%) 13 (7%)	6.5 (5.6) 70 (72%) 27 (28%) 42 (43%) 28 (29%) 19 (20%) 8 (8%)	5.3 (5.3) 219 (78%) 61 (22%) 152 (54%) 67 (24%) 40 (14%) 21 (8%)	**0.006** **0.07** **0.05**
**Satisfaction with social support:**	Mean (SD): Score > 8 Score ≤ 8	9.1 (1.3) 148 (81%) 35 (19%)	8.1 (2.1) 55 (57%) 42 (43%)	8.8 (1.7) 203 (72%) 77 (28%)	**<0.001** **<0.001**

**Abbreviations**: SD = standard deviation, PHQ-9 = the Patient Health Questionnaire-9 , GAD-7 = the Generalized Anxiety Disorder 7-item scale.

Logistic regression was used to test the significant predictors of high satisfaction (Table 4). Satisfaction with social support was the most significant predictor of high *satisfaction* with cancer care (OR: 3.8, *P <* 0.001), followed by having ≤ 4 household residents (OR: 3.3*P* < 0.001). Other significant predictors included not receiving radiotherapy (OR: 0.4, *P* = 0.008), receiving hormonal therapy (OR: 2.7, *P = 0.*04), and biological therapy (OR: 2.6, *P* = 0.04). Nationality (non-Saudi) and employment (employed) showed a strong trend toward statistical significance (OR: 0.4, *P* = 0.06 and OR: 1.8, *P* = 0.06, respectively).

**Table 4:** Logistic regression for high satisfaction (score > 8) with cancer care among patients in Saudi Arabia (N = 280).

**Variable**	**OR (95% CI)**	***P* value**
Nationality (Saudi)	0.4 (0.12–1.07)	0.06
Residency (Owned)	0.6 (0.28–1.23)	0.10
Employed	1.8 (0.95–3.51)	0.06
Household members ≤ 4	3.3 (1.6–6.6)	**<0.001**
Satisfaction with social support (score > 8)	3.8 (2.03–7.1)	***<*0.001**
Received radiation	0.4 (0.23–0.81)	**0.008**
Received hormonal therapy	2.7 (1.003–7.4)	**0.04**
Received biological therapy	2.6 (1.01–7.1)	**0.04**
Depression score > 9	0.53 (0.22–1.2)	0.15
Anxiety score > 9	1.18 (0.5–2.7)	0.60

**Abbreviations**: OR, odds ratio; CI, confidence interval.

## 4 Discussion

Patient satisfaction with cancer care is an important factor for increasing health-related quality of life (HRQoL) (Moreno et al., 2018) and patient adherence to medical recommendations (Chino et al., 2014, Hall and Dornan, 1990). The association of satisfaction with cancer care with greater HRQoL, was explained by the self-management of distress, social support, social activities, and patient-provider communication (Moreno et al., 2018). Lower satisfaction may reduce patient compliance and thereby obstruct the effective management of cancer (Becker-Schiebe et al., 2015, Nguyen et al., 2011) as well as reduce survival (Alessy and Lüchtenborg Dr, 2019).

In our sample, 65% of patients were satisfied with cancer care, which was a similar proportion as that in a Danish study (61.9%) (Heerdegen et al., 2017). However, it is difficult to compare our data with other similar literature, as they are inconsistent and heterogeneous because of differences in study designs, questionnaires, study populations, and sample sizes (Gupta, 2009).

The tools used to assess patient satisfaction have varied in the literature (Gupta, 2009). These tools include but are not limited to the European Organization for Research and Treatment of Cancer inpatient satisfaction questionnaire (EORTC QLQ-SAT32) (Bredart et al., 2004), Patient Satisfaction and Quality in Oncological Care (PASQOC) (Kleeberg et al., 2008), the Long-Form Patient Satisfaction Questionnaire (PSQ-III) (Groff et al., 2008), the Princess Margaret Hospital Satisfaction with Doctor Questionnaire (PMH-PSQ-MD) (Landen Jr et al., 2003) and the Visual Analog Scale (VAS) that we used in our study (Blanchard et al., 1990, McCormack et al., 1988, Voutilainen et al., 2016). We used the VAS because it is very brief, easy to administer and less vulnerable to bias from confounding factors and avoided the ceiling effect (Voutilainen et al., 2016).

The literature on patient satisfaction is rich. However, findings on patient-related determinants of satisfaction are markedly inconsistent in the literature (Hall and Dornan, 1990). Fox et al. stated, ‘Sociodemographic variables (SD variables) like race, age, sex, income, etc. can relate directly to satisfaction in one study, inversely in another, and be unrelated in a third. The situation has grown so chaotic that some writers dismiss SD variables as reliable predictors of satisfaction’ (Fox and Storms, 1981).

In our study, the logistic regression analysis showed that among the social factors, nationality, residency, and employment were statistically significant determinants of satisfaction. Saudis were found to be less satisfied than non-Saudis (OR: 0.4, P = 0.06). Although several studies have measured satisfaction in Saudi Arabia (Al‐Borie and Sheikh Damanhouri, 2013, Al Anazi et al., 2019, Mohamed et al., 2015), no study has compared Saudis and non-Saudis. Patients who owned their residence were more satisfied with cancer care than those who rented. Employment was also associated with greater satisfaction with cancer care (OR: 1.8, P = 0.06), which is consistent with the literature (Tang et al., 2018).

The highest independent determinant of satisfaction with cancer care in this study was satisfaction with social support (OR: 3.8, *P* < 0.001). Local and international studies suggested that patient satisfaction levels with cancer care were significantly influenced by the interpersonal aspects of care (Banaser et al., n.d., Gupta, 2009). Indeed, social support is pivotal for all patients, especially cancer patients. Social support improves patients’ coping mechanisms, decreases distress, and increases well-being and self-esteem as well as HRQoL (Dirksen, 2000, Gonzalez-Saenz de Tejada et al., 2017, Mattioli et al., 2008, Yoo et al., 2017). Social support was found to be associated with a greater sense of community and hence general satisfaction with life (Hombrados-Mendieta et al., 2019). It was shown that higher perceived social support had a positive impact on patient attitudes toward healthcare and family relationships (YILMAZ ÖZPOLAT et al., 2014). This finding emphasizes the need for social workers in every oncology setting (Thome et al., 2018).

In the other hand, we found that satisfaction with cancer care was also correlated with having<4 household residents (OR: 3.3, P = 0.0004). This finding emphasizes that an individual’s perceived social support may differ from the actual availability of social support (McDowell and Serovich, 2007). In addition, an individual may have more household residents due to having a lower socioeconomic status (SES), which in turn has been associated with dissatisfaction with health care (Chino et al., 2014).

Treatment modalities can play a role in patient satisfaction with cancer care (Carnevale et al., 2013, Nguyen et al., 2011). In our study, radiotherapy was independently associated with lower satisfaction with cancer care (OR: 0.4, P = 0.008). Similar data were shown in some other studies (Nguyen et al., 2011). Lower satisfaction with radiotherapy might be related to inaccurate information about radiotherapy and its side effects (Becker-Schiebe et al., 2015) or possible delays in treatment decisions, fears about the incorrect delivery of the treatment, and discomfort during therapy (Nguyen et al., 2011). The provision of appropriate care by radiation therapists and pain control were reported to be the main determinants of patient satisfaction (Famiglietti et al., 2013), in addition to the explanation of patients’ illnesses and radiation toxicities (Zissiadis et al., 2006).

Unlike radiotherapy, we found biological therapy (OR: 2.6, P = 0.04) and hormonal therapy (OR: 2.7, P0.04) to be independently associated with higher satisfaction with cancer care. Wood et al. observed fewer concerns about side effects and higher treatment satisfaction for biological therapy than chemotherapy (Wood et al., 2017). In addition, patients treated with hormonal therapy reported greater HRQoL, less activity impairment, and better outcomes than those treated with chemotherapy (Gupta et al., 2014).

Psychiatric disorders (anxiety and depression) were common in our study. Same was found for suicidal ideations. Also, depression and anxiety were found to be negatively correlated with patient satisfaction with cancer in univariate analysis, which is in consistent with previous literature (Lam et al., 2018). Worldwide, the prevalence of depressive spectrum disorders is 3–5 times more prevalent among cancer patients than in the general population and range between 5% and 60% according to the different diagnostic criteria, the tools used in the studies, as well as the stage and type of cancer (Caruso et al., 2017). Overall, depression and anxiety were found to be associated with a significantly increased risk of cancer incidence, cancer-specific mortality, and all-cause mortality in cancer patients (Wang et al., 2020). In addition to the classical risk factors, both biological cancer- or treatment-related mechanisms (e.g. cytokine and inflammation mechanisms, depression-inducing drugs) and psychological (e.g. coping, personality) factors, should also be considered (Caruso et al., 2017). Depression was also attributed to dissatisfaction with information given to patients, therefore, high level of information and tailoring the involvement in decision making to patients' desired level can help patients to better cope with their illness (Llewellyn et al., 2006, Vogel et al., 2009). Overall, these finding highlights the importance of screening and treating depression in every oncology setting, as depression treatment improves patient satisfaction with cancer care (Kavalnienė et al., 2018) and patient prognosis and quality of life (Wang et al., 2020).

A majority of studies found that one of the important predictors of satisfaction with cancer care is when health information about the illness and the course of treatment was comprehensively discussed with the patient .This is followed closely by the time spent with the physician, interpersonal skills of the physician, waiting time to get an appointment, empathy of staff with the patient, the continuity of care provided, satisfaction with the nursing staff as well as the management of pain and side effects, and the continuity of care (Gupta, 2009).

The current study is considered the first local study that have examined in details the impact of psychosocial variables on patient satisfaction with cancer care. Moreover, the major study variables were measured using standard validated tools. Nevertheless, a number of limitations are acknowledged. First, although, our sample was recruited from an academic medical city which provides free secondary and tertiary care services to patients from everywhere in Saudi Arabia, however, being a single-institution study, with convenient sampling, the current findings should be generalized cautiously. The cross sectional design does not ascertain causations but rather associations. Therefore, this support the need for future longitudinal studies to confirm the current findings.

## 5 Conclusions

This study found an inadequate level of patient satisfaction with cancer care. Higher levels of satisfaction were associated with being satisfied with social support, using biological and hormonal therapy, while lower satisfaction was associated with a larger number of household residents, depression, anxiety and using radiotherapy.

Declaration

## Funding

This research did not receive any specific grant from funding agencies in the public, commercial, or not-for-profit sectors.

**Ethics approval and consent to participate**

Ethical approval reference number E-17–2769 was granted on the 18th of December 2017 from the institutional review board at the Faculty of Medicine at King Saud University in Riyadh. All participants signed informed consent forms before completing the questionnaires.

## Declaration of Competing Interest

The authors declare that they have no known competing financial interests or personal relationships that could have appeared to influence the work reported in this paper.
